# Lycopene from Red Guava (*Psidium guajava* L.): From Hepatoprotective Effect to Its Use as Promising Self-Emulsifying Drug Delivery System for Anti-Inflammatory and Antioxidant Applications

**DOI:** 10.3390/ph16060905

**Published:** 2023-06-20

**Authors:** Maíra Bernardes Alves, Andreanne Gomes Vasconcelos, Amandda Évelin Silva de Carvalho, Robson Camilotti Slompo, Bruno Silva Sá, Maria Júlia Lima Gonçalves, Liz Nayara Ribeiro da Costa Lima Moura, Ana Karolinne da Silva Brito, José Vinícius de Sousa França, Maria do Carmo de Carvalho e Martins, Márcia dos Santos Rizzo, Susana Soares, Verónica Bastos, Felipe Saldanha de Araujo, Bassam Felipe Mogharbel, Katherine Athayde Teixeira de Carvalho, Helena Oliveira, Alexandra Plácido, Daniel Dias Rufino Arcanjo, Eder Alves Barbosa, José Roberto de Souza de Almeida Leite

**Affiliations:** 1 Núcleo de Pesquisa em Morfologia e Imunologia Aplicada, NuPMIA, Faculdade de Medicina, Universidade de Brasília (UnB), Brasília 70910-900, Brazil; maira-bernardes@hotmail.com (M.B.A.); andreannegv@gmail.com (A.G.V.); bioederr@gmail.com (E.A.B.); 2 Department of Biomedicine, Centro Universitário do Distrito Federal (UDF), Brasília 70390-045, Brazil; brunosilvasa13@outlook.com (B.S.S.); julia.limagon@hotmail.com (M.J.L.G.); liz.acacio@gmail.com (L.N.R.d.C.L.M.); 3 People & Science Pesquisa Desenvolvimento e Inovação LTDA, Brasília 70790-120, Brazil; felipearaujo@unb.br; 4 Laboratório de Hematologia e Células-Tronco (LHCT), Faculdade de Ciências da Saúde, Universidade de Brasília (UnB), Brasília 70910-900, Brazil; amanddaevelin@hotmail.com; 5 Instituto de Pesquisa Pelé Pequeno Príncipe, Curitiba 80240-020, Brazil; robsoncamilotti@gmail.com (R.C.S.); bassamfm@gmail.com (B.F.M.); katherinecarv@gmail.com (K.A.T.d.C.); 6 Departamento de Biofísica e Fisiologia, Centro de Ciências da Saúde (DBFis/CCS), Universidade Federal do Piauí (UFPI), Teresina 64049-550, Brazil; anakarolinnesb@hotmail.com (A.K.d.S.B.); vinicius.sfranca@ufpi.edu.br (J.V.d.S.F.); carminhamartins@ufpi.edu.br (M.d.C.d.C.e.M.); daniel.arcanjo@ufpi.edu.br (D.D.R.A.); 7 Interdisciplinary Laboratory for Advanced Materials (LIMAV), Department of Morphology, Health Sciences Center (DMOR/CCS), Federal University of Piauí (UFPI), Teresina 64049-550, Brazil; marciarizzo@ufpi.edu.br; 8 CESAM—Centre for Environmental and Marine Studies, Department of Biology, University of Aveiro, 3810-193 Aveiro, Portugal; susudade1@hotmail.com (S.S.); veronicabastos@ua.pt (V.B.); holiveira@ua.pt (H.O.); 9 Departamento de Bioquímica, Faculdade de Ciências (FCUP), Universidade do Porto (UP), 4169-007 Porto, Portugal; alexandra.nascimento@fc.up.pt; 10 Bioprospectum, UPTEC, 4200-135 Porto, Portugal

**Keywords:** lycopene, *Psidium guajava*, self-emulsifying, antioxidant, anti-inflammatory, keratinocytes, hypercholesterolemia

## Abstract

Lycopene is a carotenoid with potential use in the treatment of chronic illnesses. Here, different formulations of lycopene were studied: lycopene-rich extract from red guava (LEG), purified lycopene from red guava (LPG) and a self-emulsifying drug delivery system loaded with LPG (nanoLPG). The effects of administering orally various doses of LEG to hypercholesterolemic hamsters were evaluated regarding the liver function of the animals. The cytotoxicity of LPG in Vero cells was analyzed by a crystal violet assay and by fluorescence microscopy. In addition, nanoLPG was employed in stability tests. LPG and nanoLPG were tested for their cytotoxic effect on human keratinocytes and antioxidant capacity on cells in an endothelial dysfunction model in an isolated rat aorta. Finally, the effect of different nanoLPG concentrations on the expression of immune-related genes (*IL-10*, *TNF-*α, *COX-2* and *IFN*-γ) from peripheral blood mononuclear cells (PBMC) using real-time PCR was also analyzed. Results suggest that LEG, despite not being able to improve blood markers indicative of liver function in hypercholesterolemic hamsters, reduced hepatic degenerative changes. Additionally, LPG did not show cytotoxicity in Vero cells. In relation to nanoLPG, the effects produced by heat stress evaluated by Dynamics Light Scattering (DLS) and visually were loss of color, texture change and phase separation after 15 days without interfering with the droplet size, so the formulation proved to be efficient in stabilizing the encapsulated lycopene. Although LPG and nanoLPG showed moderate toxicity to keratinocytes, which may be related to cell lineage characteristics, both revealed potent antioxidant activity. LPG and nanoLPG showed vasoprotective effects in aortic preparations. The gene expression assay indicates that, although no significant differences were observed in the expression of *IL-10* and *TNF-α*, the PBMCs treated with nanoLPG showed a reduction in transcriptional levels of *IFN-γ* and an increased expression of *COX-2*. Thus, the work adds evidence to the safety of the use of lycopene by humans and shows that tested formulations, mainly nanoLPG due to its stability, stand out as promising and biosafe products for the treatment of diseases that have oxidative stress and inflammation in their etiopathology.

## 1. Introduction

Carotenoids are pigments recognized as bioactive compounds with positive health impacts that are unique to plants and are responsible for the bright colors of fruits and vegetables. Being the primary dietary supply of vitamin A for humans, they have provitamin and antioxidant activities [1,2]. Carotenoids are industrially used as special foods, nutraceuticals, colorants in cosmetics, animal feed additives and pharmaceuticals [3]. Moreover, carotenoids are thought to be effective against degenerative conditions, and although the mechanisms are not fully understood, this can be due in part to their antioxidant activities [4,5]. Thus, to optimize the potential of carotenoid-rich products, certain attempts have been made to design specific conditions for formulations and processes [6].

Lycopene is an uncyclized carotenoid of molecular formula C_40_H_56_ [7] with more effective singlet oxygen scavenging activity [8]. Two non-conjugated double bonds are responsible for their high antioxidant action, giving greater reactivity to the molecule [9]. Studies have shown that lycopene is essential for cellular system defense, stabilizing reactive oxygen (ROS) and nitrogen (RNS) species [10,11]. Thus, lycopene has been found to have a beneficial effect in treating chronic illnesses caused by oxidative stress, which have oxidative stress as part of their etiopathogenesis, such as cancer, obesity, cardiovascular diseases, diabetes mellitus, Alzheimer’s and inflammatory diseases [12,13,14,15]. Furthermore, studies suggest that lycopene helps to reduce the expression of genes that cause systemic inflammation and the acute phase response through modulation of interleukins and *TNF-*α synthesis in lymphocytes and macrophages, respectively [16,17]. 

Tomatoes are the traditional source of lycopene, which is being applied for the development of several products [18]. However, as the demand for lycopene is widely increasing, the exploitation of alternative resources for obtaining lycopene has become essential. In that sense, research involving red guava (*Psidium guajava* L.) as a source of lycopene has proven to be a promising choice [19], considering it provides other antioxidant carotenoids, such as β-carotene [20]. Recently, analytical methodologies for carotenoid extraction and isolation from red guava have been developed, resulting in the production of a lycopene-rich extract from red guava (LEG) and purified lycopene from red guava (LPG) [21,22]. LEG and LPG presented a greater amount of lycopene than tomatoes, exhibited antimicrobial and antioxidant activities, and inhibited carrageenan-induced acute inflammation in mice [18,23]. The beneficial effects of LEG on human breast adenocarcinoma cells [24] and decreasing blood triglycerides and lipid peroxidation in hamsters fed a high-cholesterol diet [25] were also assessed.

Like other natural carotenoids, lycopene possesses low solubility in water, is highly susceptible to light, oxygen and autooxidation, and has low intestinal permeability, challenging its clinical and food applications [26,27,28,29]. In this scenario, nanotechnology applications have emerged as promising solutions [17]. Recently, [30] developed a new LPG-based formulation by self-emulsifying drug delivery (nanoLPG). The formulation exhibited 10-month stability when stored at 5 °C, in vitro antioxidant activity, cytotoxicity towards prostate cancer cells, and the absence of toxicity in mice when orally administered.

Now, this work brings new insights into the toxicity, activity and antioxidant/anti-inflammatory mechanisms of the different formulations based on lycopene from red guava. Initially, LEG—a sample obtained using clean methodology—was assessed for liver function and toxicity in hypercholesterolemic hamsters as an inflammation and oxidative stress model. LPG and nanoLPG were assessed for cytotoxicity and vascular antioxidant effects. Then, the effects of nanoLPG on the expression of immune-related genes were also analyzed, aiming to better understand the anti-inflammatory mechanisms induced by the nanostructured LPG.

## 2. Results

### 2.1. Characterization of LEG and LPG

The spectrophotometric analysis of LEG and LPG in chloroform:ethanol (1:20) exhibited the three maximum absorption signals at 503, 472 and 447 nm expected for lycopene. The measure of lycopene content in the extract performed spectrophotometrically at 472 nm showed a yield of 20–30% and >90% of lycopene per dry extract weight for LEG and LPG, respectively.

### 2.2. Thermodynamic Stability of nanoLPG

**Figure 1:**
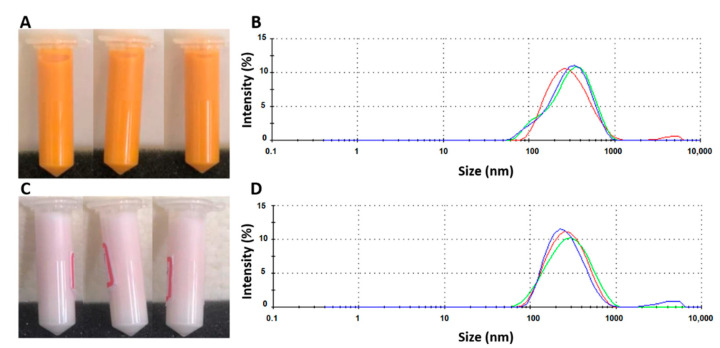
Macroscopic aspects of nanoLPG in triplicate (**A**,**C**) and droplet size distribution curve in triplicate (**B**,**D**) before (above) and after (below) thermal stress with temperatures of 4 and 45 °C every 48 h for 30 days.

The nanoLPG was subjected to physical stress, and its stability was evaluated by DLS and visual inspection. The effects of thermal stress on mean particle size, PDI, zeta potential and macroscopic aspects of nanoLPG are shown in Table 1. Results indicate that after 15 days of thermal stress, only loss of color (Figure 1A,C), change of texture (from milky liquid to creamy), and phase separation after centrifugation were observed without affecting the size of the droplets (Figure 1B,D).

### 2.3. Effect of LEG on Liver Function and Histopathological Parameters of Hypercholesterolemic Hamsters

**Figure 2:**
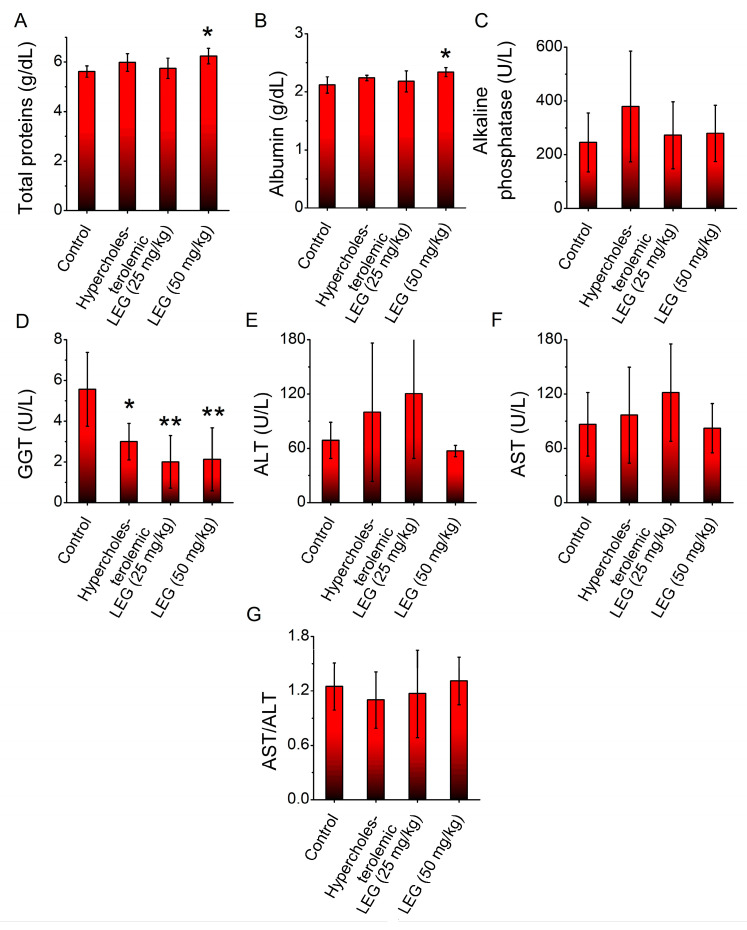
Plasma levels of (**A**) total proteins, (**B**) albumin, (**C**) alkaline phosphatase, (**D**) GGT, (**E**) ALT, (**F**) AST and (**G**) AST/ALT ratio of normal and hypercholesterolemic hamsters treated with 25 and 50 mg/kg of LEG for 28 days. Columns represent mean ± SEM; * *p* < 0.05 vs. control (vehicle) group; ** *p* < 0.01 vs. control (vehicle) group; *n* = 7–9.

Hypercholesterolemia was induced by feeding hamsters a high-cholesterol feed, according to Brito et al. [25]. The effect caused by oral administration of LEG at doses of 25 mg/kg and 50 mg/kg on blood markers indicative of liver function in the animals was measured. Results show that the high-cholesterol diet significantly decreased the plasma levels of GGT in relation to the control group (non-hypercholesterolemic hamsters) (Figure 2D). The oral administration of LEG did not prevent the decrease in the GGT level (Figure 2D). On the other hand, oral administration of LEG at a dose of 50 mg/kg significantly increased (*p* < 0.05) the plasma total proteins (Figure 2A) and albumin (Figure 2B) when compared with the vehicle group. No significant alterations were observed in the plasma alkaline phosphatases, ALT and AST.

**Figure 3:**
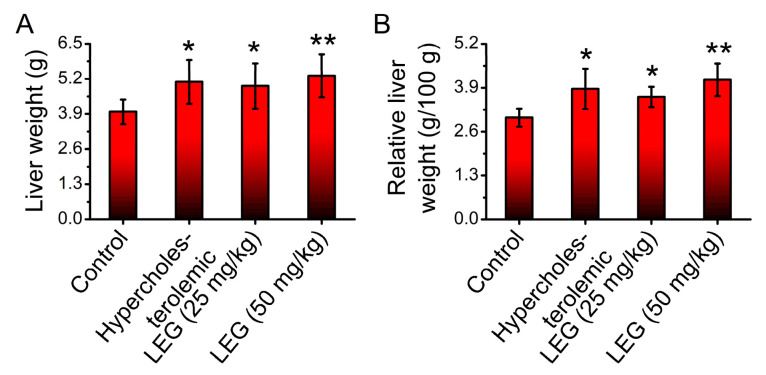
Liver weight (**A**) and relative liver weight (**B**) of normal and hypercholesterolemic hamsters treated with 25 and 50 mg/kg of LEG for 28 days. Columns represent mean ± SEM; * *p* < 0.05 vs. control (vehicle) group; ** *p* < 0.01 vs. control (vehicle) group; *n* = 7–9.

The effect caused by oral administration of LEG (25 mg/kg and 50 mg/kg) on liver weights was also evaluated. Results indicate an increase in liver weight in the hypercholesterolemic hamsters treated and non-treated with LEG when compared with the control (vehicle) group (Figure 3A). The liver/animals’ body weight ratio indicates a similar result (Figure 3B).

**Figure 4:**
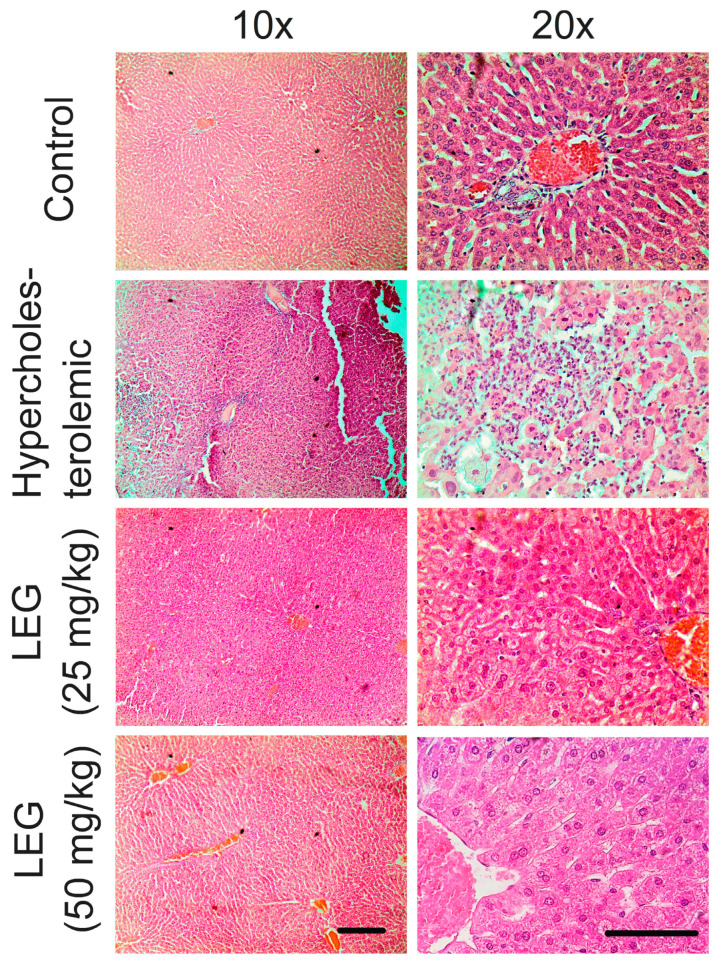
Photomicrographs of liver tissues collected from normal and hypercholesterolemic hamsters treated with 25 and 50 mg/kg of LEG for 28 days. Hematoxylin & Eosin (HE)-stained. Scale bar: 100 μm.

Histological analyses of the liver indicate that a high-cholesterol diet-induced multifocal hepatonecrosis surrounded by mononuclear inflammatory cells and moderate Kupffer cell hyperplasia. In addition, there are many small lipid droplets within some hepatocytes (microvesicular steatosis). These data are compatible with score three of the semi-quantitative analysis (Figure 4). Then, treatment with LEG at 25 and 50 mg/kg/day for 28 days significantly reduced hepatic degenerative changes (scores between one and two).

### 2.4. LPG Cytotoxicity against Vero Cells

**Figure 5:**
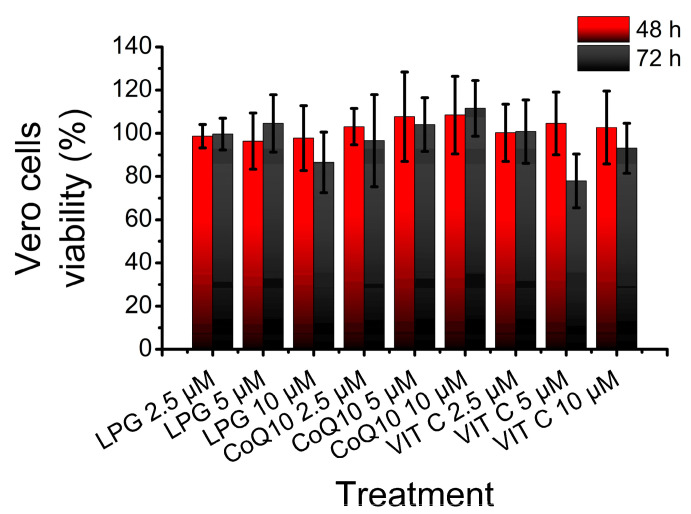
Effect of LPG, CoQ10 and VIT C on Vero Cell viability after 48 h and 72 h of exposition. Columns represent the mean ± SEM; *n* = 5.

The effect of LPG on Vero cell viability was tested by a crystal violet assay. Vero cells were treated for 48 and 72 h with LEG, CoQ10 and VIT C at increasing concentrations (2.5, 5 and 10 μM). Results show that only the treatment with VIT C at 5 μM for 72 h produced an apparent reduction of Vero cells viability, but without statistical significance (Figure 5).

**Figure 6:**
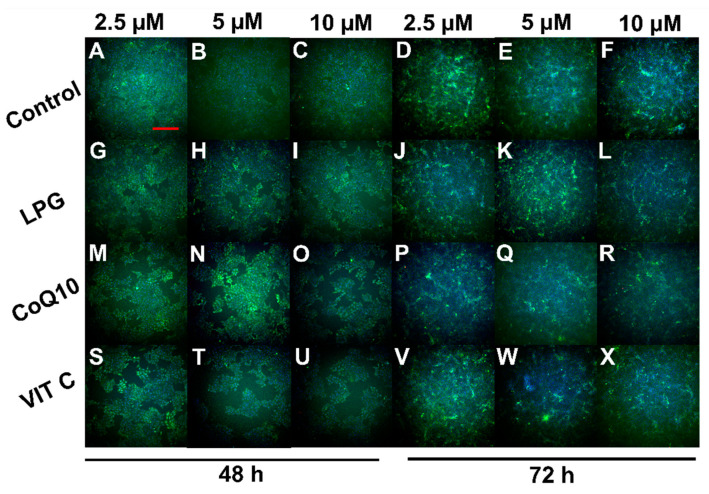
Analysis of Vero cells using Fluorescence Microscopy and the LIVE/DEAD^TM^ Viability/Cytotoxicity kit. (**A**–**F**) Control cells; Vero cells treated for 48 and 72 h with (**G**–**L**) LPG, (**M**–**R**) CoQ10 and (**S**–**X**) VIT C; red bar = 400 μm.

The cell viability results of Vero cells treated with LPG were further confirmed by fluorescence microscopy using the LIVE/DEAD^TM^ Viability/Cytotoxicity kit. This test is based on distinguishing dead and live cells according to the observed fluorescence. Living cells fluoresce green due to the activity of intracellular ubiquitin esterase, which is determined by the enzymatic conversion of non-fluorescent calcein-AM into fluorescent calcein. Dead cells are labeled with ethidium homodimer-1 (Eth-1), which crosses the damaged membrane and binds with nucleic acids, generating a bright red fluorescence. Figure 6 shows the staining profiles of control Vero cells (Figure 6A–F) and cells treated with LPG (Figure 6G–L), CoQ10 (Figure 6M–R) and VIT C (Figure 6S–X) for 48 and 72 h at concentrations of 2.5, 5 and 10 μM. In general, Vero cells stained green for all conditions tested, except for a few cells staining red (Figure 6C,H,N,R,U).

### 2.5. Cytotoxic and Antioxidant Effects of LPG and nanoLPG on Human Keratinocytes

**Figure 7:**
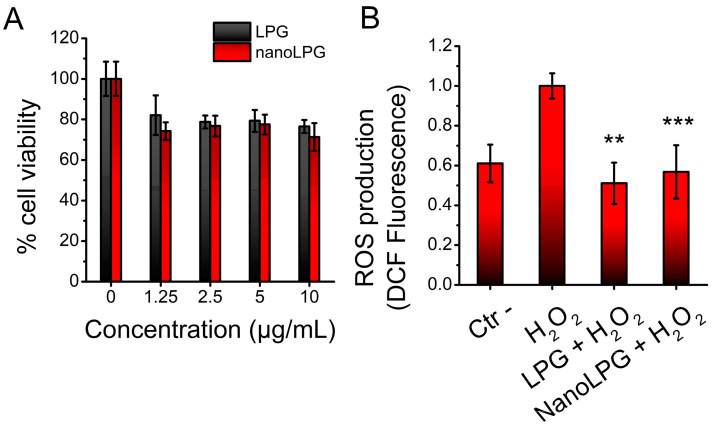
Effects of LPG and nanoLPG on HaCaT cells. (**A**) Cell viability after 24 h of exposition. (**B**) Intracellular ROS scavenging activity after cell stimulation using H_2_O_2_ at 300 μM for 30 min. Columns represent mean ± SEM; ** *p* < 0.01 vs. H_2_O_2_ group; *** *p* < 0.001 vs. H_2_O_2_ group; *n* = 5–6.

The LPG- and nanoLPG-induced cytotoxic effects at different concentrations on human immortalized keratinocytes (HaCaT cells) were tested by MTT assays. Results show a reduction in keratinocyte viability of 17.9 ± 9.7% and 25.8 ± 4.3% when the lowest concentrations of LPG and nanoLPG (1.25 μg/mL) were used, respectively (Figure 7A). Interestingly, even at 10 μg/mL, that decrease did not change significantly, so there was a reduction of 23.5 ± 3.2% and 28.7 ± 6.9% when LPG and nanoLPG were tested, respectively. The IC_50_ could not be determined.

The ability of LPG and nanoLPG to eliminate intracellular ROS induced by H_2_O_2_ (300 μM) was tested in HaCaT cells. Results show that at a concentration of 10 μg/mL, both LPG and nanoLPG inhibited ROS formation in such a way that no significant difference was observed for the negative control (Figure 7B).

### 2.6. Vascular Antioxidant Effects of LPG and nanoLPG on Pyrogallol-Induced Endothelial Dysfunction in Isolated Rat Aorta

**Figure 8:**
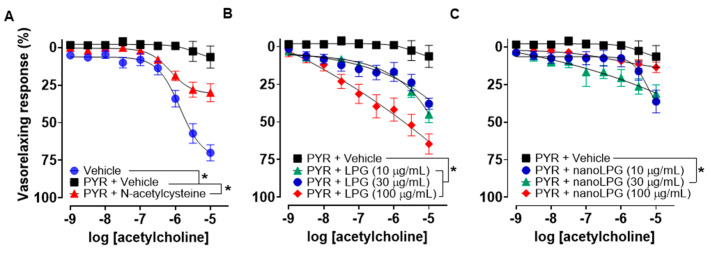
Antioxidant effect of LPG and nanoLPG on pyrogallol-induced endothelial dysfunction in the rat thoracic aorta. A) Concentration–response curves for ACh in the presence of vehicle, pyrogallol (PYR, 3 × 10^−5^ M) and N-acetylcysteine (3 × 10^−5^ M) followed by PYR. Effects of LPG B) or nanoLPG C) followed by PYR on ACh-induced concentration–response curves. Dots represent mean ± SEM; * *p* < 0.05 vs. vehicle; *n* = 6–7.

The antioxidant activities of LPG and nanoLPG were tested on endothelium-intact aortic rings incubated with the superoxide generator pyrogallol. In the presence of pyrogallol, the ACh-induced vasorelaxant response was markedly abolished (Figure 8A). On the other hand, pre-incubation with N-acetylcysteine was able to partially inhibit the pyrogallol-induced endothelial dysfunction (Figure 8A). In another set of experiments, the pre-incubation of LPG at 100 μg/mL was able to induce complete vascular protection against the effects of pyrogallol, whereas concentrations of 10 and 30 μg/mL promoted partial protection similar to that observed for N-acetylcysteine (Figure 8B). Besides, nanoLPG was able to promote vascular protection similar to that of N-acetylcysteine only at a concentration of 30 μg/mL and solely at the highest concentration of ACh at a concentration of 10 μg/mL (Figure 8C). No vascular antioxidant effect for nanoLPG was observed at 100 μg/mL.

### 2.7. Effects of nanoLPG on the Expression of Immune-Related Genes in Human Peripheral Blood Mononuclear Cells (PBMCs) of Health Donors

**Figure 9:**
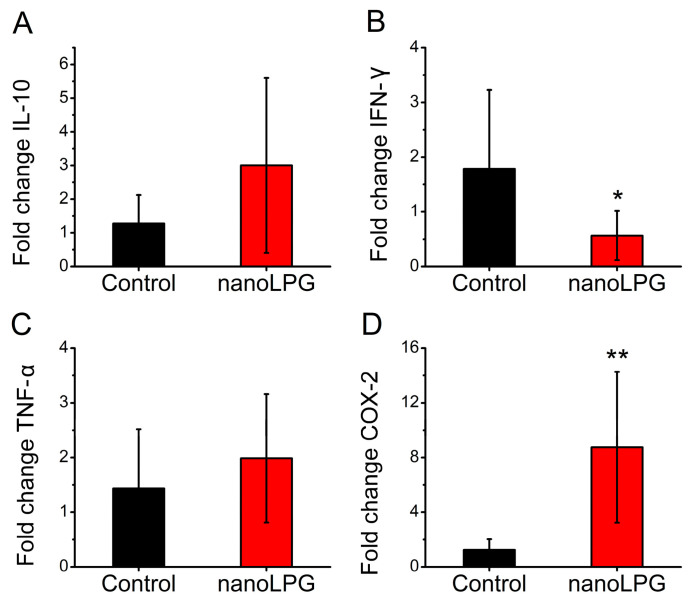
Relative gene expressions of (**A**) *IL-10*, (**B**) *IFN*-γ, (**C**) *TNF-*α and (**D**) *COX-2* in PBMCs after treatment with nanoLPG (25 μg/mL) for 24 h, compared with non-treated cells (control). Columns represent mean ± SEM; * *p* < 0.05 vs. control group; ** *p* < 0.005 vs. control group; *n* = 3.

Peripheral blood samples from healthy donors were subjected to density gradient procedures aimed at the isolation of PBMCs. Total RNA from mononuclear cells untreated and treated with nanoLPG (25 μg/mL) was isolated, quantified and subjected to reverse synthesis. The obtained cDNA was used as a template for real-time PCR reactions to quantify the expression of genes of interest using the *GAPDH* gene as a normalizer. Results show that, while there was no significant difference in the expression of *IL-10* (Figure 9A) and *TNF-*α (Figure 9C), PBMCs treated with nanoLPG showed a reduction in transcriptional levels of *IFN*-γ (Figure 9B) and increased expression of *COX-2* (Figure 9D).

## 3. Discussion

This study performed both in vivo and in vitro experiments to evaluate the roles of lycopene in LEG, LPG and nanoLPG formulations on inflammation and oxidative stress models. According to previous studies, the production of these formulations was successful, and spectrophotometric analysis at 472 nm confirmed that LEG and nanoLPG, respectively, yielded 20–30% and >90% of lycopene per dry extract weight [30,31].

The thermodynamic stability tests performed for nanoLPG show loss of color, change of texture (from milky liquid to creamy), and phase separation after centrifugation. Although the loss of color may be related to lycopene degradation, it occurred after 15 days of thermal stress by exposing nanoLPG to 45 °C. Thus, the lycopene in the formulation remained stable for 14 days, while Lee and Chen [32] reported a decrease in the total lycopene content after 12 h at 50 °C. The creamy aspect observed may be related to water evaporation, thus increasing the concentration of surfactants present in the nanoLPG formulation. The phase separation is usually caused by processes such as aggregation, creaming, sedimentation, coalescence and flocculation [33]; however, the occurrence of such processes was not evidenced by DLS analysis. Additionally, the phase-separated samples restored homogeneity in a few seconds after agitation. Overall, nanoLPG proved to be stable during the tests, corroborating the potential of the formulation for improving lycopene stability [30].

The lycopene extract from guava (LEG) is a sample obtained by an efficient, economical and safe approach, even though its lycopene yield is lower. To evaluate its effects on liver function in hypercholesterolemic hamsters, which experience increased inflammation and oxidative stress due to their cholesterol-rich diet [34,35], LEG was initially tested. Although LEG treatment did not reverse the blood markers indicative of liver function, histopathological analyses indicated a decrease in hepatic degenerative changes, suggesting a protective effect against inflammatory injury. These results are in agreement with previous studies that reported no systemic toxicity when mice were given lycopene from red guava [30] and beneficial effects on dyslipidemia and oxidative stress [25]. The potential mechanisms through which LEG protects the liver from inflammatory injury may involve its ability to inhibit the production of pro-inflammatory cytokines and modulate apoptotic pathways. Lycopene extracted from red guava has been demonstrated to possess anti-inflammatory effects, including the inhibition of leukocyte mobilization, the stabilization of mast cells, and the suppression of genes that are connected to inflammation [23]. In addition, lycopene has also been shown to modulate apoptosis pathways, which play an important role in liver injury. Indeed, a recent study demonstrated that lycopene was able to inhibit apoptosis in the liver by reducing the expression of apoptosis-related proteins [36]. 

Given that LPG has a higher lycopene content than LEG and is not allowed for application in food due to its potential cytotoxicity, its toxicity was initially evaluated. Lycopene has been deemed safe for use by humans and other animals, even in large doses [37,38]. In this study, LPG displayed cytotoxicity in keratinocytes of the HaCaT cell line at relatively low concentrations, resulting in around a 20% decrease in cell viability. However, further investigation is necessary as the use of a non-primary cell line may have impacted the results, as Vero cells viability was not affected by the LPG treatment. Additionally, nanoLPG-induced cytotoxicity on HaCaT cells was similar to that of free LPG and to that described by Vasconcelos et al. [30], where nanoLPG was not significantly cytotoxic to PBMCs after 24 h at concentrations ranging from 0.75 to 25 µg/mL.

The antioxidant activities of LPG and nanoLPG on DPPH and ABTS free radical scavenging methods were previously reported [30,39]. In the present work, LPG and nanoLPG also show potent antioxidant activity, preventing intracellular ROS formation on human keratinocyte cells stimulated with H_2_O_2_. It is well known that ROS induce cell apoptosis [40,41,42], and antioxidants can counteract apoptosis in keratinocytes induced by H_2_O_2_ [43]. Indeed, the protective effects of lycopene on skin cells have been reported [27,44,45,46,47]. Thus, the formulations tested, mainly nanoLPG due to its stability, show high potential for use as a skin protective product. In addition, LPG was able to promote a marked vasoprotective effect by improving the acetylcholine-induced vasorelaxant effect in aortic preparations with pyrogallol-induced oxidative endothelial dysfunction. Although nanoLPG presented a lower effect when compared with LPG, this effect was even similar to that of N-acetylcysteine, a well-known antioxidant sulfhydryl donor. In this sense, both LPG and nanoLPG were able to promote a vasoprotective effect against oxidative endothelial damage. A decrease in mortality related to coronary heart disease, cerebrovascular disease, atheroscl, erosis and congestive heart failure has been linked to lycopene-based supplementation [48,49]. In this sense, lycopene has been demonstrated to act as a vasoprotector, promoting an improvement of endothelial function and a reduction of atherosclerotic plaques and vascular dementia, where anti-inflammatory and antioxidant mechanisms underlying those effects have been demonstrated [50,51,52].

To understand the anti-inflammatory mechanisms of the guava lycopene nanostructured product, immune-related gene expression in human peripheral blood mononuclear cells after treatment with nanoLPG was performed. NanoLPG modulated the gene expression of PBMCs by decreasing *IFN*-γ and increasing *COX-2* gene expression. The immunomodulatory effect of lycopene on non-stimulated PBMCs from healthy individuals [53,54,55] and on different cell types in a proinflammatory state induced by LPS, mainly, has been described [56,57,58,59,60,61]. In this work, we perform experiments on untreated cells from healthy donors. Although the effect of nanoLPG on *IFN*-γ expression in our study agrees with the literature for free LPG [53,54], LPS-stimulated cells treated with lycopene usually show a decrease in *COX-2* gene expression [56,58,62]. It is important to mention that the effect of lycopene on the generation of inflammatory mediators has been shown to have a wide range of variations [53,54,56,57,58,59,60,61]. Even though the increase in *COX-2* expression was surprising, it is important to emphasize that nanoLPG appears to modulate the Th1 response by not only suppressing the transcription of *IFN*-γ but also elevating the transcriptional levels of the anti-inflammatory factor *IL-10*. Moreover, further research is necessary to determine the effect of nanoLPG on the *COX-2* expression of non-treated PBMCs, including the use of varying concentrations, as lycopene has been demonstrated to induce either an inflammatory or anti-inflammatory state in CaCo-2 cells depending on the dose [60].

## 4. Material and Methods

### 4.1. Lycopene-Rich Extract from Red Guava (LEG), Purified Lycopene from Red Guava (LPG) and the Self-Emulsifying Drug Delivery System Loaded with Purified Lycopene from Red Guava (nanoLPG)

#### 4.1.1. Samples

LEG production was carried out according to patent no. EP3400812 [22]. LPG was produced according to the methodology described in the patent document nº BR 102016030594-2 [21]. LEG and LPG were obtained from red guavas at a high degree of maturation, obtained from organic cultivation in the Tabuleiros Litorâneos do Piauí (Parnaíba, Piauí, Brazil), and acquired from a local market. The fruits were heated, ground, and mixed with solvents. For LEG, the extraction was performed with analytical-grade ethanol (Dinâmica, Rio de Janeiro, Brazil) under ultrasonic stirring. For LPG, the extraction procedure was performed using analytical-grade dichloromethane (Scharlab S. L., Barcelona, Spain) under ultrasonic stirring, followed by crystallization at −20 °C, purification with chloroform (Sigma-Aldrich Chemical Co., St. Louis, MO, USA) through quantitative filter paper, and storage at −80 °C after obtention. The LEG and LPG contents were determined by spectrophotometric analysis [30,31]. NanoLPG was produced according to Vasconcelos et al. [30] using sorbitan monostearate, coconut oil, ethanol, acetone, distilled water (pH 7.0) and polysorbate 80. The synthesis conditions were carried out under magnetic stirring at 40 °C for 10 min and solvent evaporation (rotavap) at reduced pressure (30 mBar) and a temperature of 37 °C. The samples were stored at 5–8 °C after obtention.

#### 4.1.2. Characterization of nanoLPG

Mean particle size (z-average) and size distribution (polydispersity index—PDI) were determined by Dynamics Light Scattering (DLS) using a Zetasizer Nano-ZS90 (Malvern Panalytical Ltd, Malvern, UK), as described by Vasconcelos et al. [30]. Briefly, samples were diluted in ultrapure water for both measures, then analyzed in triplicate at a 90° angle with an equilibration time of 60 s at 25 °C prior to measurement. The same conditions were used to determine the zeta potential.

#### 4.1.3. Thermodynamic Stability of nanoLPG

The nanoemulsion was subjected to physical stress to evaluate its stability, as described by Azeem et al. [63]. Samples (2.0 mL) were centrifuged at 3500 rpm for 30 min and 15,000 rpm for 15 min. For the thermal stability assay, a sample (2 mL) was heated (45 °C) and cooled (4 °C) at 48-hour intervals for 30 days. Color, texture, phase separation and turbidity were macroscopically evaluated. Mean particle size, zeta potential and PDI were analyzed in accordance with the method above-mentioned. The test was carried out in triplicate.

### 4.2. Blood Markers Indicative of Liver Function and Histopathological Study in Hypercholesterolemic Hamsters Treated with LEG

#### 4.2.1. Ethical Aspects

All animal procedures were conducted in accordance with the ethical standards established by the National Council for the Control of Animal Experimentation (CONCEA, Brazil) and by Brazilian Laws (11,794, of 10.8.2008 and Law 9605, of 02.12.98). The experimental protocols were previously submitted to the Ethics Committee for the Use of Animals of the Universidade Federal do Piauí (Teresina, Brazil) and approved under document no.CEUA-UFPI Nº 197/16.

#### 4.2.2. Animals

Male hamsters (*Mesocricetus auratus,* Golden Syrian strain), 16 days old and weighing 53.26 ± 1.99 g, were housed in individual cages with free access to feed and water, under a 12/12 h light–dark cycle, and at a controlled temperature (23 ± 2 °C). Dyslipidemia was induced for 21 days in accordance with Brito et al. (2019), while the control animals (normolipidemic) received a normolipidemic diet (standard rodent feed; Labina, São Paulo, SP, Brazil) until the end of the experiment. Subsequently, the animals underwent 28 days of treatment, divided into the control (standard rodent feed), hypercholesterolemic (high cholesterol feed), LEG-25 (high cholesterol feed + LEG orally at 25 mg/kg/day), and LEG-50 (high cholesterol diet + LEG orally at 50 mg/kg/day) groups. At the end of the experiment, the animals were euthanized with an intraperitoneal dose of sodium thiopental (100 mg/kg per body weight) and lidocaine (10 mg/kg). For the separation of the blood plasma, venous blood samples were centrifuged at 2500 rpm for 15 min at 4 °C.

#### 4.2.3. Effect of LEG on Liver Function

Plasma levels of total proteins, albumin, alkaline phosphatase, gamma-glutamyltransferase (GGT), aspartate aminotransferase (AST) and alanine aminotransferase (ALT) were determined using an automatic analyzer and reagent kits according to the manufacturer’s methodology (Labmax Plenno, Labtest, Lagoa Santa, MG, Brazil). The De Ritis ratio, or AST/ALT ratio, was calculated as described by De Ritis et al. [64].

#### 4.2.4. Effect of LPG on Morphological Changes of the Liver

The livers were collected and fixed in 10% buffered formalin for 48 h to subsequently undergo the usual histological routine, which comprises dehydration, diaphanization, impregnation with paraffin, blockage, microtomy and deparaffinization. Sections (5 μm thick) were stained with hematoxylin–eosin (HE), followed by evaluation using a light microscope (Olympus Microscope, Tokyo, Japan). The histopathological analysis was performed blindly, according to the semiquantitative classification criterion, on a scale of scores ranging from 0 to 4, as stated by Farias et al. [65].

### 4.3. LPG Cytotoxicity Assays on Vero Cells

#### 4.3.1. Cell Culture

Vero cells (Rio de Janeiro Cell Bank, Rio de Janeiro, Brazil; BCRJ code: 0245) were removed from the liquid nitrogen and incubated in a water bath at 37 °C for a short period. As soon as the contents of the flask began to thaw, they were transferred into a 15-milliliter tube that contained complete culture medium (DMEM-F12 supplemented with 10% FBS (fetal bovine serum) and 1.0% Penicillin (100 U/mL) and Streptomycin (100 μg/mL) solutions). For 10 min, the tube was centrifuged at 1500 rpm. The supernatant was subsequently removed, and then a complete culture medium was added to the cells. An aliquot was taken for cell counting, performed with trypan blue (1:1 dilution) in a Neubauer chamber. After counting, cells were seeded at 1 × 10^4^ cells/cm² in a 75 cm^2^ culture flask. Culture flasks were kept in an incubator at 37 °C and 5% CO_2_. The culture medium was replaced twice a week until the cells were subcultured or used for the experiments.

#### 4.3.2. Cristal Violet Assay

Vero cells (2 × 10^3^ cells/well) were seeded in 96-well plates. After 24 h, the supplemented DMEM-F12 was replaced by culture media containing the treatments (LPG, coenzyme Q10 (CoQ10) and ascorbic acid (VIT C) at 2.5, 5 and 10 μM). After 48 and 72 h, the culture medium was withdrawn, and cells were then washed three times with PBS (physiological buffer solution). Then, 200 μL of 2.5% glutaraldehyde was added, and the plates were maintained at room temperature. After 3 h, the glutaraldehyde solution was removed, and then the cells were washed with PBS once. Then, 200 μL of crystal violet was added, and cells were maintained at room temperature. After 10 min, the crystal violet was withdrawn, and cells were washed twice with Milli-Q water, followed by the addition of 200 μL of 33% glacial acetic acid. Finally, the plates were shaken for 30 min, and absorbances were measured at 595 nm. Vero cells treated with the respective solvents without LPG, CoQ10 or VIT C were submitted to the same procedures and used as controls.

#### 4.3.3. Evaluation of Vero Cell Viability by the LIVE/DEAD^TM^ Viability/Cytotoxicity Kit

After 24 h of previous seeding of Vero cells (5 × 10^3^ cells/well) in 96-well plates, the supplemented DMEM was replaced by a culture medium containing the treatments (LPG, coenzyme Q10 (CoQ10) and ascorbic acid (VIT C) at 2.5, 5 and 10 μM). After 48 and 72 h, each well received the addition of Hoechst 33342, Calcein-AM and ethidium homodimer-1 at concentrations of 5 μg/mL, 0.3 nM and 0.6 nM, respectively. The plates were incubated and protected from light for 30 min and analyzed using an IN Cell Analyzer 2000 device (GE Healthcare, Buckinghamshire, UK), where 4 images per well were captured. Vero cells treated with the respective solvents without LPG, CoQ10 or VIT C were submitted to the same procedures and used as controls.

### 4.4. Cytotoxic and Antioxidant Effects of LPG and nanoLPG on Human Keratinocytes

#### 4.4.1. Cell Culture

Immortalized human keratinocyte cells (HaCaT) were acquired from Cell Lines Services (Eppelheim, Germany), and cultured in supplemented DMEM containing 10% FBS, 1.0% L-glutamine, fungizone and penicillin–streptomycin (Life Technologies, Grand Island, NY, USA), at 37 °C in a humidified atmosphere at 5.0% CO_2_. Cell morphology was analyzed using a Nikon Eclipse 80i inverted microscope (Nikon, Tokyo, Japan).

#### 4.4.2. MTT Assay

The colorimetric (3-(4,5-dimethylthiazol-2-yl)-2,5-diphenyltetrazolium bromide) MTT assay was used to evaluate the cytotoxic effects of LPG and nanoLPG. Briefly, HaCaT cells (6 × 10^3^ cells/well) were seeded and allowed to adhere in 96-well plates. Then, cells were treated with a range of concentrations of LPG and nanoLPG (1.25; 2.5; 5 and 10 μg/mL) diluted in DMEM medium for 24 h at 37 °C in 5% CO_2_. After exposure time, 50 μL of MTT solution (Sigma-Aldrich, 1 mg/mL in PBS, pH 7.2) was added to each well, and the plate was incubated for 4 h. After incubation, the medium was removed, dimethyl sulfoxide (DMSO, 150 μL) was added to dissolve the formazan crystals, and the plate was shaken for approximately 2 h under protection from light. The optical density of reduced MTT was measured at 570 nm using a microplate reader (Synergy HT from BioTeK Instruments Inc., Winooski, VT, USA) to determine the cell viability. The ratio of the absorbance of treated cells to control cells was used to calculate the proportion of viable cells.

#### 4.4.3. Intracellular ROS Scavenging Activity

The intracellular ROS levels in HaCaT keratinocytes were evaluated by the 2′,7′-dichlorodihydrofluorescein diacetate (DDCF-DA) assay. For this, keratinocytes were seeded in 12-well plates (6 × 10^4^ cells/well), and after cell adherence, they were treated with LPG and nanoLPG at 10 μg/mL for 24 h. Cells were then exposed to H_2_O_2_ at 300 μM for 30 min and then incubated for 30 min with 10 μM DCFH-DA. After that, cells were detached, and DCF fluorescence was analyzed within 45 min on an Attune^®^ Acoustic Focusing Cytometer (Applied Biosystems, Life Technologies, Carlsbad, CA, USA). The mean fluorescence intensity (MFI) of DCF was used to estimate ROS formation using FlowJo software (Tree Star Inc., Ashland, OR, USA).

### 4.5. Effects of LPG and nanoLPG on Pyrogallol-Induced Endothelial Dysfunction in Isolated Rat Aorta

#### 4.5.1. Ethical Aspects

All animal procedures were performed following ethical standards established by the National Council for the Control of Animal Experimentation (Brazil) and by Brazilian Laws (11,794, of 10.8.2008 and Law 9605, of 02.12.98). The Ethics Committee for the Use of Animals of the Universidade Federal do Piauí (Teresna, Brazil) has previously appreciated the experimental protocols and has approved them under document CEUA-UFPI No. 457/18.

#### 4.5.2. Animals

Female Wistar rats (180–250 g, 8–12 weeks old) were obtained from the Animal Facility of the Universidade Federal do Piauí (Teresina, PI, Brazil). The animals were maintained with free access to feed and water under a 12/12 h light–dark cycle at a controlled temperature (23 ± 2 °C) throughout the experiments.

#### 4.5.3. Preparation of Aortic Rings and Vascular Reactivity

The aortic preparations were performed according to Arcanjo et al. [66]. In order to assess whether the LPG and nanoLPG induce vascular antioxidant effects, endothelium-intact aortic rings were incubated with the superoxide generator pyrogallol (30 μM) for 30 min after pre-incubation with LPG or nanoLPG (10, 30 and 100 μg/mL) or N-acetylcysteine (NAC, 3 × 10^−5^ M), an antioxidant sulfhydryl donor, for 30 min. Then, phenylephrine (3 × 10^−7^ M) was added, and at the tonic phase of contraction, ACh (10^−9^–10^−5^ M) was cumulatively added to obtain a concentration–response curve [67,68] by non-linear regression and statistical significance by a two-way ANOVA followed by Bonferroni’s post-hoc test using GraphPad Prism 8.0 (GraphPad Inc., La Jolla, CA, USA).

### 4.6. Effect of nanoLPG on Immune-Related Gene Expression in Human Peripheral Blood Mononuclear Cells (PBMCs)

PBMCs were incubated with nanoLPG at different concentrations, and expressions of the following immune-related genes were assessed by real-time PCR (qPCR): *IL-10*, *TNF-*α, *COX-2* and *IFN*-γ. For this, the total RNA of the cells submitted to incubation was extracted and submitted to cDNA synthesis. This cDNA was then used in real-time PCR experiments using specific oligonucleotides for the analyzed genes.

#### 4.6.1. Obtaining Human PBMCs

PBMCs were isolated by density gradient using Ficoll-Paque (Amersham Biosciences, Amersham, UK). Human peripheral blood samples were collected from three healthy donors in EDTA tubes. Whole blood (4 mL) and PBS buffer (4 mL) were transferred to conical tubes loaded with Ficoll-Paque (3 mL) and centrifuged at 360× *g* for 25 min. Then, mononuclear cells were obtained and washed three times with PBS. Subsequently, lysis of erythrocytes was performed with 10 mL of 0.8% NH_4_Cl in 0.1 mM EDTA (*v*/*v*). After 10-min homogenization, tubes were centrifuged for 10 min at 250× *g* and 4 °C. Two washing cycles with PBS containing 2.0% FBS were performed, and the resulting cell pellet was dispersed in 1.0 mL of RPMI medium supplemented with 10% FBS. The cell count was carried out in a Neubauer chamber.

#### 4.6.2. Exposition of Human PBMCs to nanoLPG, RNA Extraction and cDNA Synthesis

Human PBMCs (1 × 10^6^ cells/well) were plated in RPMI medium containing 10% FBS and then incubated with 25 μg/mL of nanoLPG. After 24 h, cells were harvested and subjected to RNA extraction (GenElute™ Mammalian Total RNA Miniprep Kit, Sigma-Aldrich, St. Louis, MO, USA). Nanodrop One spectrophotometer (ThermoFisher Scientific, Waltham, MA, USA) was used to verify the quantity and quality of the total RNA. For cDNA synthesis, total RNA (500 ng) and the High-Capacity cDNA Reverse Transcription Kit (ThermoFisher Scientific, Waltham, MA, USA) were used.

#### 4.6.3. Real-Time PCR

Quantitative analyses of gene expression were performed using TaqMan methodology (ThermoFisher Scientific, Waltham, MA, USA), using primers and probes acquired by the AssayOnDemand system. The reactions were carried out in duplicates using the following conditions: 2 min at 50 °C and 10 min at 95 °C (holding stage), followed by 40 cycles of 15 s at 95 °C and 1 min at 60 °C. Relative gene expression was determined by the 2^−ΔΔCt^ method [69,70]. To normalize the samples, cycle threshold (ΔCt) differences were calculated by subtracting the Ct value of the internal reference (GAPDH) from the Ct values of the assessed genes. The mean ΔCt values of non-treated PBMCs (the control group) were used as a reference. The following genes were analyzed using GoTaq^®^ Probe qPCR Master Mix (Promega, Madison, WI, USA): *IL-10* (Hs00961622) and *TNF-*α (Hs01113624). The SYBR Green methodology was used to analyze the expression of *COX-2* and *IFN*-γ genes using specific primers (Table 2). The StepOne Plus real-time PCR instrument (Thermo Fisher) was used for analysis.

### 4.7. Statistical Processing

Mean ± standard error of mean (SEM), and the statistical significance was evaluated by two-way ANOVA using OriginPro 8.5 for graph plotting. For experiments involving pyrogallol-induced endothelial dysfunctions in isolated rat aortas, the mean ± SEM percentage of vasorelaxation as a function of the logarithm of cumulative acetylcholine concentrations was used to obtain concentration–response curves. These curves were plotted using non-linear regression, and statistical significance was determined by a two-way ANOVA among the experimental groups (vehicle, LPG and nanoLPG). Dunnett’s multiple comparisons post-test was employed to assess the between-group significance for each cumulative acetylcholine concentration. Both graph plotting and statistical analysis were carried out using GraphPad Prism 8.0 software (GraphPad Inc., La Jolla, CA, USA).

## 5. Conclusions

Although LEG was not able to improve blood markers indicative of liver function in hypercholesterolemic hamsters, it protected against hepatic degenerative changes and showed no cytotoxicity to Vero cells. The nanoLPG formulation was found to be thermodynamically stable, preserving the physical–chemical properties of lycopene, which are responsible for its biological activities, such as its antioxidant activity, demonstrated by its scavenging of intracellular ROS when keratinocytes were induced with H_2_O_2_ and its modulation of the expression of *IFN*-γ and COX-2 genes. This study provides evidence for the safety of lycopene for use in humans and animals, as well as the potential of LPG and nanoLPG to act as vasoprotective agents in aortic preparations in the presence of a superoxide generator. Overall, nanoLPG stands out as a promising and biosafe tool for the treatment of diseases involving oxidative stress and inflammation, including cardiovascular and other dysfunctional endothelium-related diseases. Future research directions could involve investigating the potential health benefits of lycopene formulations and their effect on specific diseases/conditions. In terms of potential applications, lycopene formulations, especially nanoLPG, could be tested as dietary supplements to promote health and wellness or for medical purposes, such as the treatment of certain diseases and conditions.

## Figures and Tables

**Table 1:** The effects of thermal stress on mean particle size, PDI, zeta potential and macroscopic aspects of nanoLPG.

Parameter	No Stress (Day 0)	No Stress (Day 60)	Thermal Stress (Day 30)
**Size (nm)**	258.60 ± 4.65	263.00 ± 5.76	243.30 ± 3.51
**PDI**	0.22 ± 0.00	0.23 ± 0.01	0.19 ± 0.02
**Zeta Potential (mV)**	−39.20 ± 1.46	−34.10 ± 1.27	−37.60 ± 1.27
**Macroscopic aspect**	Intense orange-coloured, turbid, milky liquid, homogeneous	Intense orange-coloured, turbid, milky liquid homogeneous	Weaker colour, turbid, creamy, phase separation

**Table 2:** Sequence of primers used for Real-time PCR.

Gene	**Forward**	Reverse
*COX-2*	GAAGTTGGCAGCAAATTGAGC	TTCTCCTGTGAAGGCGATGA
*IFN*-γ	ACTGTCGCCAGCAGCTAAAA	TATTGCAGGCAGGACAACCA

## Data Availability

Data is contained within the article.
